# Association between Influenza vaccination and severity of lung involvement at CT images of the patients with COVID-19 infection: an Iranian retrospective case-control study

**DOI:** 10.22088/cjim.13.0.221

**Published:** 2022

**Authors:** Marzihe Faraji, Rahele Mehraeen, Mehrdad Nabahati, Mahmoud Sadeghi Haddad Zavareh, Hoda Shirafkan, Yousef Yahyapour, Ghazal Mohamadi

**Affiliations:** 1 Department of Radiology, Babol University of Medical Sciences, Babol, Iran; 2 Non-Communicable Pediatric Disease Research Center, Health Research Institute, Babol University of Medical Sciences, Babol, Iran; 3 Infectious Diseases and Tropical Medicine Research Center, Health Research Institute, Babol University of Medical Sciences, Babol, Iran; 4 Social Determinants of Health Research Center, Health Research Institute, Babol University of Medical Sciences, Babol, Iran; 5 Tehran University of Medical Sciences, Tehran, Iran

**Keywords:** COVID-19 (corona virus disease) Infection, Influenza Vaccination, Lung CT scan, Severity

## Abstract

**Background:**

Frequent waves of corona virus disease (COVID-19) and lack of specific drugs against that, warrant studies to reduce the morbidity and mortality of this pandemic disease. In this study, we investigated the association between influenza vaccination and the severity and outcome of COVID-19 disease in Iranian patients living in the North.

**Methods:**

This retrospective case-control study was performed on186 patients with COVID-19 infection between March and April, 2020. Patients with positive PCR were divided into two groups of case and control; Patients with moderate to severe and normal to mild lung involvement, respectively. The lung opacities in all of the 5 lobes were evaluated on chest CT images using a CT severity scoring system. The history of influenza vaccination during the fall of 2019-2020 was determined by a phone call. Statistical analysis was done using the chi-square test, student’s t-test, and logistic regression. The significance level was p<0.05.

**Results:**

The mean age of patients was 54.67±15.05years. Most patients had pulmonary manifestations including ground-glass opacity (57%), consolidation (80%) and pleural effusion (3.2%). Adjusting for age, gender, and history of underlying disease, vaccination is an effective factor in the severity of pulmonary involvement (AOR=0.39; 95%CI: (0.21, 0.73); P=0.003). Furthermore, the chance of ICU admission decreased via influenza vaccination (OR=0.21, P=0.001).

**Conclusion:**

The results showed that the severity of COVID-19 pulmonary involvement and outcome as ICU admission, and severe symptoms in patients with history of influenza vaccination were significantly lower than those without history of vaccination. This strategy can be used to prevent and reduce the complications of COVID-19.

COVID-19 virus infection has started in China since 2020 and has spread quickly in the world, and the World Health Organization declared this global pandemic as a public health emergency in January 2020 (1). In Iran, the first case of this virus was reported on February 19, 2020, and so far, over 1.6 million people were definitively diagnosed with the infection and nearly 60,000 people died (until February 25, 2021) (2). COVID-19 infection covers a wide range of symptoms, from mild cold symptoms to acute respiratory syndrome and death, and most involvements are associated with the respiratory system, with fever, cough, and shortness of breath (3). The definitive diagnosis of COVID-19 disease is made by reverse transcription-polymerase chain reaction (RT-PCR), but the sensitivity of this laboratory test is not enough (4). CT scan of the lung is also used in rapid diagnosis and clinical decision (5), and its sensitivity in identifying this disease is reported to be 97% (6).

Most imaging findings of CT scan regarding lung infection caused by COVID-19 were reported as ground-glass opacity (GGO) and interstitial changes, with peripheral and multifocal distribution (6). Some studies reported that vaccination is a factor that affects the severity of COVID-19 infection. Salem M.L et al. (2020) reported that influenza vaccination is associated with a reduction in mortality and disease severity in patients with COVID-19 virus (7). Fink, G et al. (2020) and Marín-Hernández et al. also confirmed these results (8, 9). However, Ragni et al. (2020) stated that vaccination is not an effective factor in reducing the severity of COVID-19 disease and mortality in people under 65 years of age (10). Some researchers have reported that influenza vaccination may worsen the prognosis (11). The basic mechanisms of the protective role of influenza vaccination on the outcomes of COVID-19 are not exactly known. Immunological and epidemiological studies (12-15), as well as mathematical models (16, 17) indicate that vaccination against one microorganism may affect the host's response to other infectious agents. For example, vaccination against mycobacterium tuberculosis with Bacillus Calmette-Guérin (BCG) vaccine significantly increases the secretion of IL-1B (proinflammatory cytokine), which plays an important role in antiviral immunity (18). 

Recognition of influenza vaccination and COVID-19 disease severity and outcome relationships can be useful in disease prevention and improve prognosis. This study was performed to assess the effect of influenza vaccination on the severity of lung involvement in patients with confirmed COVID-19 disease. The effect of influenza vaccination on other consequences of COVID-19 disease (mortality and ICU admission) were considered as secondary objectives.

## Methods

**Study design and samples: **This retrospective case-control study was performed on patients with COVID-19 that referred to Ayatollah Rouhani Hospital - a referral center for care of patient with COVID-19 infection in Babol, North of Iran- between March and April, of 2020. The protocol of this study was approved by the Ethics Committee for Research of Babol University of Medical Sciences (IR.MUBABOL.REC.1399.442). The data from the medical records were extracted and reviewed by two radiologists with at least 12 years’ experience in reporting lung CT scan. All patients with COVID-19 whose diagnosis was confirmed by nasopharyngeal/oropharyngeal mucosal real time- PCR were included in the study. Based on lung CT scan features and using a scoring system, the patients were divided into two groups: Patients with positive PCR and moderate to severe lung involvement (case group) and patients with positive PCR and mild to normal lung involvement (control group).

The history of influenza vaccination during the fall of 2019-2020 was determined in each group by a phone call. Informed consent was obtained from all patients at the beginning of the telephone call. The vaccine used in the study population was influvac quadrivalent vaccine (Netherlands).

**Evaluation of severity of lung involvement: **Scoring was done by CT scan at the time of diagnosis of patients based on the following criteria: Presence of ground-glass opacity, presence of consolidation, number of lobes affected by ground-glass or consolidative opacities, degree of lobe involvement in relation to overall lung as “total severity score”. 

Each lung lobe was then scored separately according to the following criteria (table 1): (19).

**Table 1:** CT severity score index

**Involvement of lung**	**Score**
None (0%),	0
Minimal (1%–25%),	1
Mild (26%–50% )	2
Moderate (51%–75%),	3
Severe (76%–100%)	4

**Collected data: **Demographic information (including age and gender), medical history (including cardiopulmonary disease, hypertension, diabetes), severity of lung involvement, ICU admission, multidrug treatment and mortality rate were recorded. 

**Statistical analysis: **Descriptive statistics were used as mean ± standard deviation (SD) for quantitative variables and frequency (percentages) for qualitative variables. To compare participants' demographic and medical history characteristics between case and control groups, we used chi-square test (or Fisher exact test) and student’s t-test.  In addition, to assess the effect of influenza vaccine on severity of lung involvement adjusting for potential confounding variables (such as age, gender, DM, hypertension, and heart diseases) we used multivariable logistic regression. Crude and adjusted odds ratio (AOR) and their 95% confidence interval (95%CI) were calculated. Statistical analyses were performed using SPSS (Version 22). The significance level was considered as p< 0.05.

## Results

**Demographic and clinical findings: **The mean age of patients was 54.67±15.05 and there was a significant difference between the two groups (P=0.011). Approximately, 60% of patients were males. About half of patients had at least a history of diseases such as diabetes (20%), hypertension (17.8%), heart disease (24.7%), lung disease (1.1%), kidney disease (0.6%), and malignancy (0.6%). A significant statistical change between the groups in terms of diabetes, hypertension, and heart disease was seen. But there was no statistical difference in history of lung disease between two groups (table 2). In addition, we compared the participant's basic characteristics between two groups of with and without influenza vaccination (table 3). 

**Table 2:** Demographic characteristics and medical history in patients with COVID-19 infection

**Characteristics a**		**Overall**	**Group**		**P-value**
			**severe to moderate lung involvement (case)**	**mild to normal** **lung involvement (control)**	
Age (Year) (Mean ± SD)		54.67±15.05	57.45±15.95	51.89±13.61	0.011 b
Age	≤50	80 (43.0)	32 (34.41)	48 (51.61)	0.018 c
	>50	106 (57.0)	61 (65.59)	45 (48.39)	
Sex	Male	113 (60.75)	60 (64.52)	53 (56.99)	0.29 c
	Female	73 (39.25)	33 (35.48)	40 (43.01)	
Diabetic Mellitus	No	148 (79.57)	68 (73.12)	80 (86.02)	0.029 c
	Yes	38 (20.43)	25 (26.88)	13 (13.98)	
Hypertension d	No	152 (82.16)	70 (75.27)	82 (89.13)	0.014 c
	Yes	33 (17.84)	23 (24.73)	10 (10.87)	
Heart Diseases	No	140 (75.27)	61 (65.59)	79 (84.95)	0.002 c
	Yes	46 (24.73)	32 (34.41)	14 (15.05)	
History of lung disease	No	184 (98.92)	93 (100)	91 (97.85)	0.497 e
	Yes	2 (1.08)	0	2 (2.15)	
ground-glass opacity	No	80 (43.00)	61	19	<0.001
	Yes	106 (57.00)	32	74	
consolidation d	No	37	1	36	<0.001 e
	Yes	148	92	56	
pleural effusion	No	180 (96.78)			
	Yes	6 (3.22)			

a: Values are presented as mean ± SD and frequency (percentage). b: Independent t-test c: Chi-square test. d: There is one missing. e: Fisher exact test Significant P-values are shown as bold text.

**Relationship between influenza vaccination and severity of lung involvement**: Most patients had pulmonary manifestations including ground-glass opacity (57%), consolidation (80%) and pleural effusion (3.2%). Comparisons of types of lung involvements between cases and controls as well as with/without vaccination are shown in tables 2 and 3. In the five categories of pulmonary involvement, 8 patients had no lung involvement, 25 patients were in group minimal, 35 patients were in group mild, 64 patients in group moderate and finally 54 patients were categorized as severe lung involvement. A statistically significant difference was seen between the two groups of vaccination (P=0.005). By dividing pulmonary involvement into two categories of mild and severe, 49 (26.3%) patients were in the mild group and 137 (73.7%) patients were in the severe group. Univariable logistic regression showed that influenza vaccination, age, history of DM, hypertension, and heart disease were the potential factors affecting severity of lung involvement. In other words, the chance of having moderate to severe lung involvement in patients with influenza vaccination was lower than patients without influenza vaccination. Also, with increasing one year of age, the chance of moderate to severe lung involvement increases by 26% (OR=1.26, P=0.013). Furthermore, the chance of moderate to severe lung involvement in patients with a history of DM, hypertension, and heart disease were almost twice as high as in other patients. Evaluating the effect of influenza vaccination on severity of lung involvement, after adjusting for age, gender, and history of underlying disease (diabetes, etc.), the odds of having severe lung involvement in patients with history of vaccination was lower than patients without history of vaccination (odds ratio: 0.39; 95% CI (0.21, 0.73); P=0.003), which indicates that vaccination is an effective factor in the severity of pulmonary involvement (table 4).

**Relationship between vaccination and disease outcome: **Of the 186 patients included in the study, 32 (17.2%) patients were admitted to the ICU and 28(15%) patients died. To assess the effect of influenza vaccination on ICU admission as well as mortality, we used multivariable logistic regression. Age, gender, and underling disease (DM, HTN and heart diseases) were adjusted as confounding factors. As it was seen in table 5, the vaccination is a protective factor for admission at the ICU. The chance of ICU admission decreased 0.79 times since influenza vaccination (OR=0.21, P=0.001). Furthermore, considering the effect of vaccination on mortality, vaccination reduced the chance of death but this was not significant (OR=71, P=0.41) (table 5)

**Table 3:** Comparisons of basic characteristics of patients in vaccination groups

**Characteristics ** ^a^		**With vaccination**	**Without vaccination**	**P value**
Age (Year) (Mean ± SD)		54.12±15.02	55.22±15.14	0.62 ^b^
Age	≤50	48 (30.4)	71 (43.9)	**0.032** ^ c^
	>50	79 (50.0)	73 (42.7)	
Sex	Male	57(61.3)	56(60.2)	0.88^ c^
	Female	36(38.7)	37(39.8)	
Diabetic Mellitus	No	79 (84.9)	69 (74.2)	0.069^ c^
	Yes	14 (15.1)	24 (25.8)	
Hypertension ^d^	No	80 (87.0)	72 (77.4)	0.090^ c^
	Yes	12 (13.0)	21 (22.6)	
Heart Disease	No	67 (72.0)	73 (78.5)	0.30^ c^
	Yes	26 (28.0)	20 (21.5)	
History of lung disease	No	91 (48.4)	93 (51.6)	0.497^e^
	Yes	2 (64.5)	0 (35.5)	

a: Values are presented as mean ± SD and frequency (percentage).

b: Independent t-test

c: Chi-square test.

d: There is one missing.

e: Fisher exact test

Significant P-values are shown as bold text.

**Table 4:** Association between influenza vaccination and severity lung involvement

Univariable Analysis				Multivariable Analysis		
**Variables**	**crude OR**	**P-value **	**CI (95%)**	**adjusted OR**	**P-value**	**CI (95%)**
Influenza Vaccination	0.36	0.001	0.20-0.66	0.39	**0.003**	0.21-0.73
Age (Year)	1.26	0.013	1.005-1.05	1.01	0.274	0.99-1.04
Sex (Male /Female)	1.372	0.294	0.76-2.47	1.33	0.394	0.69-2.54
Diabetic Mellitus	2.26	0.032	1.07-4.76	1.30	0.545	0.56-3.03
Hypertension	2.69	0.016	1.20-6.04	1.58	0.333	0.62-4.03
Heart Diseases	2.96	0.003	1.45-6.03	2.00	0.90	0.89-4.47

Significant P-values are shown as bold text

**Table 5:** Association between influenza vaccination and patient outcomes

**patient outcomes**	Crude OR (95% CI) Crude OR (95% CI)	P-Value	Adjusted OR (95% CI)*	P-Value
ICU	0.22 (0.09, 0.54)	0.001	0.21 (0.08-0.54)	0.001
Death	0.84 (0.37-1.89)	0.68	0.71 (0.30-1.67)	0.412

The final multivariable models were adjusted for the age, sex, Diabetic Mellitus, Hypertension, and Heart Diseases. Significant P-values are shown as bold text.

## Discussion

The aim of this study was to evaluate the association between influenza vaccination with severity of pulmonary involvement (by CT scan) in patients with COVID-19. The results of the present study showed that the severity of pulmonary involvement and the consequences of COVID-19 infection in patients with influenza vaccination were significantly lower than those without history of vaccination after adjusting the intervention variables. The frequency of symptoms (moderate to severe), hospitalization in ICU and mortality were considered as outcomes of COVID-19 infection. The results showed that the influenza vaccination reduced the chance of ICU admission as well as chance of mortality. The results of this study were in line with the studies of Pawlowski et al. (2020) (20), Fink, G et al. (2020), Amato et al. (2020) and Marín-Hernández et al. (2020) (8, 9, 21). Fink, G et al. in a study on 92,664 Brazilian patients with COVID-19 showed that the need for hospitalization in the ICU, mechanical ventilation and death in patients with a history of influenza vaccination was significantly lower than in patients without a history of vaccination (8). In another study, Pawlowski et al. reviewed the immunization records of 137,730 patients with COVID-19(PCR positive) and reported that vaccination in the past 1, 2, and 5 years was associated with a reduction in SARS-CoV-2 infection (20).

The possible mechanism for the reduction in the severity of COVID-19 disease in patients with a history of vaccination can have several justifications. The first possible justification is that evolutionarily speaking, the influenza virus is close to the SARS-CoV-2 virus and they have some common epitopes and mechanisms, (22) and common structure (23), which can elicit similar immune and hormonal responses when exposed to influenza virus and COVID-19 infection (24-26). As a result, influenza vaccination can be associated with increased immunity (7) and partial protection against COVID-19 infection (22). In these cases, researchers use the term "heterologous immunity" in which an infection by one pathogen can induce or alter the immune response to another unrelated pathogen. Another possible justification is the rate of influenza vaccination in people with higher socioeconomic status of the community, who usually have better health and awareness, and are more likely to use methods to prevent COVID-19 disease, such as using a mask and maintaining good health. Yancy CW et al. reported that low socioeconomic status is a risk factor for COVID-19 infection (27). The results of the present study were inconsistent with the studies of Ragni et al. (2020) (10) and Wolff GG et al. (2020) (28). Ragni et al. in Italy evaluated the association of influenza vaccination in 4882 patients with COVID-19. The results of their study showed that vaccination is not an effective factor in reducing the consequences of COVID-19 disease (hospitalization and death rate). It seems that the inconsistency between two studies was for most of the vaccinated COVID-19 patients in their study were older and had co-existing diseases, and both of which are known to be associated with poor outcomes in COVID-19- patients Moreover, in the study of Wolf et al. (2020) in the United States, the vaccination status of 2880 people with non-influenza respiratory viruses was compared with 3240 people with negative results (non-infected with non-influenza respiratory viruses). In their study, the risk of infection with other respiratory viruses was not different in people with and without a history of influenza vaccination (28). Furthermore, in the study of Sundaram et al. (2012), vaccination had no effect on the incidence of viral disease in children and adults (29). Some researchers believe that the reaction created by the innate immune system may not occur in interactions between viruses of different families or species (29). They also mentioned the time interval between vaccination and infection to other viral diseases and the prevalence of different viral species in different populations.

This study had several limitations. In this study, the time interval between influenza vaccination and COVID-19 disease was not evaluated, and we could gain more accurate information by measuring it. Furthermore, information related to vaccination was gathered through self-report and phone call, which may lead to bias. This was a retrospective study, but designing cohort studies can help us better understand the causal relationship between influenza vaccination and the severity and outcomes of COVID-19 disease.

In conclusion the results of the present study showed that the severity of pulmonary involvement and the outcomes of COVID-19 infection (by pulmonary CT features [mild to severe] and hospitalization in ICU in patients with history of influenza vaccination was significantly lower than those without history of vaccination. This strategy can be used to prevent and reduce the complications of COVID-19.
